# Reconfigurable symmetry-broken laser in a symmetric microcavity

**DOI:** 10.1038/s41467-020-14861-5

**Published:** 2020-02-28

**Authors:** Qi-Tao Cao, Ruishan Liu, Heming Wang, Yu-Kun Lu, Cheng-Wei Qiu, Stefan Rotter, Qihuang Gong, Yun-Feng Xiao

**Affiliations:** 1 State Key Laboratory for Mesoscopic Physics and Frontiers Science Center for Nano-optoelectronics, School of Physics, Peking University, Beijing, 100871 China; 2 Department of Electrical and Computer Engineering, National University of Singapore, Singapore, 117583 Singapore; 3 Institute for Theoretical Physics, Vienna University of Technology (TU Wien), Vienna, A-1040 Austria EU; 4 Collaborative Innovation Center of Quantum Matter, Beijing, 100871 China; 5 Collaborative Innovation Center of Extreme Optics, Shanxi University, Taiyuan, 030006 China; 6 Peking University Yangtze Delta Institute of Optoelectronics, Nantong, 226010 Jiangsu China

**Keywords:** Microresonators, Nanophotonics and plasmonics, Nonlinear optics

## Abstract

The coherent light source is one of the most important foundations in both optical physics studies and applied photonic devices. However, the whispering gallery microcavity, as a prime platform for novel light sources, has the intrinsically chiral symmetry and severely rules out access to directional light output, all-optical flip-flops, efficient light extraction, etc. Here, we demonstrate a reconfigurable symmetry-broken microlaser in an ultrahigh-Q whispering gallery microcavity with the symmetric structure, in which a chirality of lasing field is empowered spontaneously by the optical nonlinear effect. Experimentally, the ratio of counter-propagating lasing intensities is found to exceed 160:1, and the chirality can be controlled dynamically and all-optically by the bias in the pump direction. This work not only presents a distinct recipe for coherent light sources with robust and reconfigurable performance, but also opens up an unexplored avenue to symmetry-broken physics in optical micro-structures.

## Abstract (web summary)

The directional lasing emission in whispering gallery microcavities typically resorts to breaking the structure symmetry. Here the authors demonstrate a reconfigurable symmetry-broken microlaser in a symmetric ultrahigh-Q whispering gallery microcavity, in which a chirality of lasing fields is empowered spontaneously by nonlinear effects.

## Introduction

Coherent light sources lie at the heart of photonic integrated circuits and of fundamental physics^1–5^. Considerable attention in this context has been dedicated to ultrahigh-*Q* optical microcavities that have become indispensable in numerous high-performance coherent light sources with high efficiency^6–8^. As a prominent platform, whispering-gallery-mode (WGM) microcavities, benefiting from ultrahigh-Q factors and small mode volumes, have witnessed significant development of on-chip light sources ranging from orbital-angular-momentum lasers to soliton microcombs^9–14^. However, the intrinsic chiral symmetry of WGMs microcavity geometry and the resulting equivalence between the two directions of light propagation in the cavity prevent many opportunities to further applications of microlasers, such as directional light emission^15,16^, all-optical flip-flops^17,18^, and nonreciprocal light propagation^19–21^.

Existing solutions to acquire the unidirectionality of the laser propagation in an ultrahigh-Q microcavity are based on the explicit chiral-symmetry breaking of the micro-structures through gain-loss modulated system with ${\mathcal{PT}}$-symmetry^13,14^, a shape deformation of the cavity^15,16^, or an asymmetric scattering boundary^22–25^. The scalability and tunability of these preceding strategies are strongly limited, because the devices once fabricated impose a prefixed chirality to the lasing mode and cannot meet the ever-increasing demands of dynamically controlling the directionality and chirality of the laser. For example, the controllable optical bistability of the lasing direction can be used for an optical flip-flop^17,18^, and the balanced counter-propagating wave is also of importance for, such as, single nanoparticle detection^26,27^ or dual frequency comb^28^. Thus, the controllable switch between the unidirectional and bidirectional emission is promising for the multiplex photonic devices. One proposed solution is to work with a selectively pumped Brillouin laser, but satisfying the involved phase-matching condition poses a formidable challenge for any experimental implementation of this concept^20,21^. Beyond the strategies by explicit symmetry breaking, the spontaneous symmetry breaking (SSB) of the counter-propagating optical field has been demonstrated recently in a passive microcavity^29,30^, which holds potential to dynamically switch the direction of the propagating light. Nevertheless, it is still elusive for the SSB of a laser field in an active microcavity due to the accompanying complex lasing dynamics. Moreover, the emission direction would also present randomness brought by the spontaneity of the lasing chirality, posing obstacles for the controllable reconfigurability.

In this work, we start from a symmetric WGM microcavity and experimentally realize a symmetry-broken Raman laser by extending the fundamental concept of SSB into an active microcavity. The chiral symmetry of the lasing emission is broken spontaneously by the microcavity-enhanced optical Kerr nonlinearity. This SSB of the Raman laser occurs above a threshold of a few tens of microwatts, and the ratio of counter-propagating emission intensities exceeds 160:1. Moreover, the chirality can be all-optically and dynamically controlled by a bi-directional pump, and the threshold power of the SSB is adjustable using a nanotip scatterer.

## Results

### Theoretical model

**Fig. 1:**
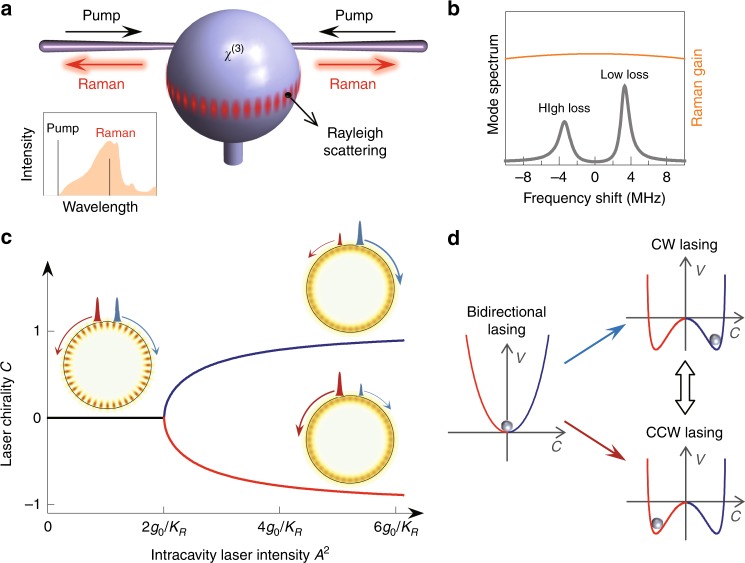
Schematic of the spontaneously symmetry-broken microlaser. **a** Illustration of the Raman laser in a microsphere, where the intracavity counter-propagating laser fields are coupled through linear Rayleigh scattering and nonlinear Kerr phase modulation. Inset: Schematic optical spectra of a Raman laser process. The orange shade denotes the Raman gain curve. **b** Mode competition of two Raman supermodes induced by the linear intrinsic coupling, where the gray curve is the Raman supermodes spectrum, and yellow curve represents the nearby Raman gain. Here only the high-frequency mode can lase due to the lower mode loss. **c** Laser chirality vs. intracavity Raman laser intensity, exhibiting a pitchfork bifurcation with the corresponding visualization of the laser-field distribution. **d** Dependence of the effective potential *V* on the laser chirality ${\mathcal{C}}$. The red (blue) branch denotes the CCW (CW) chiral laser.

The microlaser operates based on stimulated Raman scattering in a WGM microcavity (as shown in Fig. 1a)^31–33^. The corresponding Raman waves in clockwise (CW) and counterclockwise (CCW) directions are intrinsically coupled and form two standing-wave supermodes by the inevitable Rayleigh scattering arising from the roughness of the surface^34,35^. Each of the two resulting supermodes emits in both directions with equal CW and CCW intensity because of the chiral symmetry. However, only one supermode with the larger net gain will survive and consequently lase due to the mode clamping (shown in Fig. 1b and “Methods”)^33^. In addition, the CW and CCW Raman waves are also nonlinearly scattered by the optical Kerr effect, in the form of self- and cross-phase modulation^29–31^. Correspondingly, in the CW-CCW wave basis, the coupled-mode equation describing the evolution of Raman waves reads (see Supplementary Note 1),

1 $$\frac{{\rm{d}}{a}_{{\rm{R}},{\rm{m}}}}{{\rm{d}}t} \,=	\,-\frac{\kappa }{2}{a}_{{\rm{R}},{\rm{m}}}+({G}_{{\rm{R}},{\rm{m}}}| {a}_{{\rm{P,m}}}{| }^{2}+{G}_{{\rm{R}},{\rm{m}}^{\prime} }| {a}_{{\rm{P}},{\rm{m}}^{\prime} }{| }^{2}){a}_{{\rm{R}},{\rm{m}}}\\ 	+\, {\rm{i}}{g}_{0}{a}_{{\rm{R}},{\rm{m}}^{\prime} }+{\rm{i}}{K}_{{\rm{R}}}(| {a}_{{\rm{R}},{\rm{m}}}{| }^{2}+2| {a}_{{\rm{R}},{\rm{m}}^{\prime} }{| }^{2}){a}_{{\rm{R}},{\rm{m}}},$$

where *m* and $m^{\prime}$ ($m^{\prime}\;\ne\;m$) represent CW and CCW directions, ${a}_{{\rm{R}},{\rm{m}}({\rm{m}}^{\prime} )}$ (${a}_{{\rm{P}},{\rm{m}}({\rm{m}}^{\prime} )}$) represents the Raman (pump) mode, *g*_0_ is the intrinsic coupling between the counter-propagating Raman waves from the Rayleigh scattering; *κ* is the decay rate of the passive Raman cavity mode, ${G}_{{\rm{R}},{\rm{m}}({\rm{m}}^{\prime} )}$ denotes the Raman gain coefficient, and *K*_R_ is the coefficient of Kerr nonlinearity (Supplementary Note 1). If not indicated otherwise, we will assume to work with an ideal medium, where ${G}_{{\rm{R}},{\rm{m}}}={G}_{{\rm{R}},{\rm{m}}^{\prime} }$ due to the balanced forward and backward stimulated Raman scattering.

As suggested by Eq. (1), the coupling between CW and CCW Raman waves consists of both the linear Rayleigh scattering and the nonlinear scattering by the Kerr effect, resulting in an intensity-dependent effective coupling. When the nonlinearity is turned off, the Raman laser circulates bidirectionally in the cavity as a conventional standing-wave field (Fig. 1c). As the nonlinear scattering strength increases with higher laser power, the effective coupling can be reduced to zero with a particular phase condition, which corresponds to that the linear coupling is completely compensated by the nonlinear scattering^36^. Note that this condition can only be reached for the supermode with the higher frequency^29^. Above this threshold, the chiral symmetry of the laser field spontaneously breaks and the original standing-wave laser mode evolves into either one of two chiral states with a CW- or CCW-dominated laser propagation. This threshold Raman intensity of the chiral lasing is derived in Methods, and shown by Fig. 1c. The resulting laser chirality ${\mathcal{C}}$ is conveniently quantified by the order parameter ${\mathcal{C}}\equiv ({P}_{{\rm{cw}}}-{P}_{{\rm{ccw}}})/({P}_{{\rm{cw}}}+{P}_{{\rm{ccw}}})$ as defined through the Raman power *P*_cw(ccw)_ in the two counter-propagating directions. The physical nature of the emergence of SSB can be visually revealed through the effective potential *V* with a characteristic dependence on the chirality ${\mathcal{C}}$ (Fig. 1d), where the steady-state is found at the minimum of the potential curve (see the detailed derivation in the “Methods” section). Below the SSB-threshold this potential only features a single minimum with a bidirectional lasing mode and no chirality. Beyond the SSB-threshold this minimum splits into two such that the original standing-wave supermode becomes unstable. In this bistable regime either one of the two chiral minima can be populated, in principle, but we will show in detail below how to selectively initialize a desired chiral mode through the external pump bias, resulting in a dynamically switchable directionality of the laser.

### Experimental realization

**Fig. 2:**
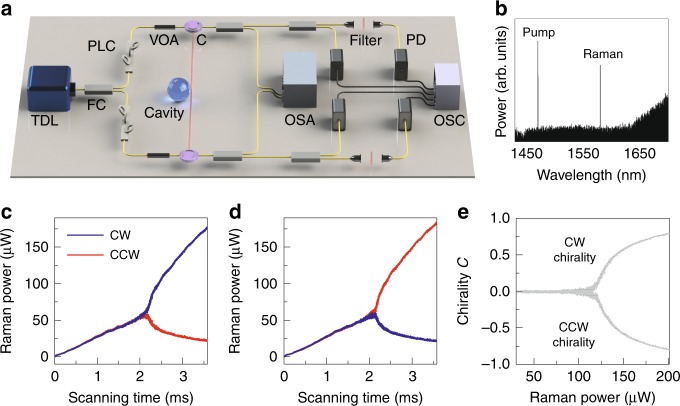
Experimental set-up and observation of a symmetry-broken Raman microlaser. **a** Illustration of experimental set-up. TDL tunable diode laser, OSA optical spectrum analyzer, PLC polarization controller, C optical circulator, PD photodetector, VOA variable optical attenuator, FC fiber coupler, OSC oscilloscope. **b** Optical spectra of the pump light and Raman laser. **c**, **d** CW (CCW) Raman output power *P*_cw_ (*P*_ccw_) vs. frequency-sweeping time. As the scanning time increases, the intracavity pump power becomes stronger, leading to chiral emission with CW (**c**) or CCW directionality (**d**). **e** Dependence of the chirality ${\mathcal{C}}$ on the total Raman power, exhibiting a balanced pitchfork bifurcation.

In the experiment, a silica microsphere resonator, with a diameter of around 30 μm and a Q factor of over 6 × 10^7^, is coupled with a tapered fiber, as illustrated in Fig. 1a and 2a. By continuously pumping in the 1470 nm band with power of 2 mW at room temperature, the Raman lasing in the 1570 nm band is excited through stimulated Raman scattering (Fig. 2b). Here neither cascaded Raman scattering nor multimode lasing occurred. The Raman output waves are separated from the pump signal by longpass filters, and the output Raman power is monitored in both directions on an oscilloscope. To demonstrate SSB with a fully symmetric system setting, the pump mode is excited on both sides with identical power and polarization, as shown in Fig. 2a. The emergence of a symmetry-broken microlaser is depicted in Fig. 2c, d. The tunable laser scans through the pump mode at a speed of  −2.2 THz/s in the blue-detuned region. As the scanning time grows, the frequency detuning between the input light and the pump mode decreases, resulting in an increasing intracavity pump power. Right above the Raman lasing threshold, the CW and CCW output Raman powers first grow synchronously with increasing intracavity pump power. Such a bidirectional Raman emission confirms that the chiral symmetry remains intact at this stage. At a sweeping time of 2.1 ms, a total Raman power of 114 μW is reached, where the chiral symmetry of the Raman laser breaks spontaneously. In the chiral regime above these values, the CW (CCW) Raman power experiences a continuous growth, while the CCW (CW) Raman power is suppressed simultaneously, leading to a CW (CCW) chiral laser in Fig. 2c (Fig. 2d). The dependence of chirality ${\mathcal{C}}$ on Raman power exhibits a balanced pitchfork bifurcation behavior, as plotted in Fig. 2e. Note that here the direction of chirality is notably random, i.e., either CW or CCW, due to the spontaneous mechanism and the fully symmetric setting of the system.

**Fig. 3:**
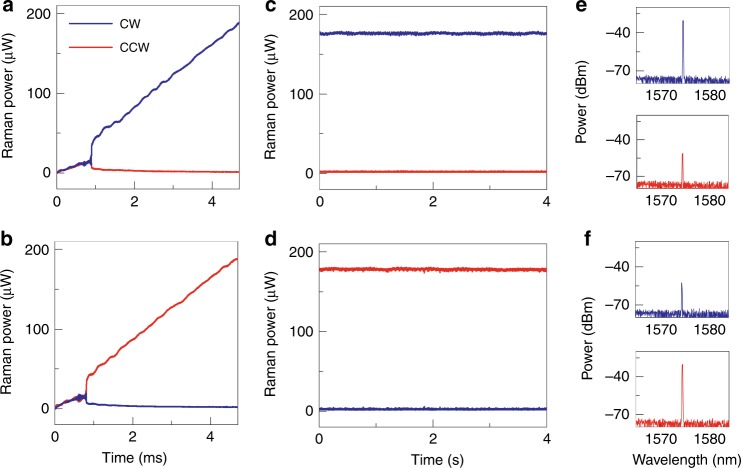
Stable Raman lasers with high chirality. **a**, **b** Similar to Fig. 2c, d but with a different pump mode, exhibiting higher chirality and lower threshold power. **c**, **d** The chiral Raman laser emits stably at stationary states when the pump laser is self-locked in the mode, resulting in a fixed intracavity pump light power. **e**, **f** The optical spectra of the Raman laser emitted in CW and CCW directions, where the resolution of the spectrometer in measurement is 0.04 nm.

The chiral behaviors of the spontaneously symmetry-broken lasers vary quantitatively from sample to sample because of the different *Q* factors, mode volumes, intrinsic coupling strengths, etc. Among the different modes for pump, a symmetry-broken Raman laser with a very low chirality-threshold power is obtained, accompanied by a larger chirality ${\mathcal{C}}$. The intensity ratio of the CW (CCW) laser to CCW (CW) laser reaches beyond 160:1 with an absolute chirality of 0.988 and a threshold Raman power of 30 μW, as shown in Fig. 3a, b. Here the increasing chirality ${\mathcal{C}}$ is bounded by the emergence of higher-order and multi-mode Raman lasing. The stability of the symmetry-broken Raman laser is further experimentally validated with a fixed-wavelength pump. By employing the thermal self-lock technique^37^, the stable CW and CCW chiral Raman emissions are recorded for 4s in Fig. 3c, d, respectively, excluding that this behavior is just of transient nature. The optical spectra of the emissions from the two directions also exhibit stable unidirectionality with high contrast, as shown in Fig. 3e, f.

**Fig. 4:**
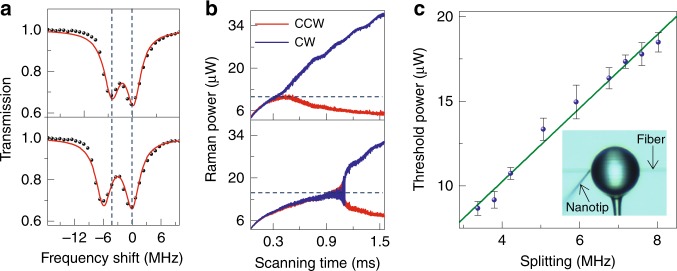
Symmetry-breaking threshold. **a** Transmission spectra of Raman modes with a smaller (top) and a larger (bottom) splitting controlled by a side-coupled scatterer, respectively. The black dots (red curves) are the measured (fitted) results, manifesting the two standing-wave supermodes featured by the double lorentzian dip. **b** Raman emission in CW and CCW directions with a smaller (top) and a larger (bottom) Raman mode splitting controlled by a side-coupled scatterer, respectively. **c** The threshold power for Raman chirality exhibits a linear dependence on the splitting in the Raman modes, where the green line is the fitting result. The error bars represent the standard deviation of repeated measurements. Inset: A silica nanotip (left side) is placed in the evanescent field of a microsphere to change the Rayleigh scattering strength.

As aforementioned, the chiral symmetry breaking emerges when the nonlinear scattering by the Kerr nonlinearity cancels out the intrinsic linear coupling, corresponding to the threshold of Raman intensity being 2*g*_0_*/K*_R_ (see the “Methods” section). We now demonstrate that the proportionality between the threshold Raman intensity and the intrinsic coupling strength *g*_0_, as predicted above, is directly accessible in the experiment (Fig. 4). In the corresponding measurements, a fiber nanotip (inset of Fig. 4c) is used as a Rayleigh scatterer to control the intrinsic coupling strength, determined by the volume of the nanotip within the evanescent field of the cavity mode^38^. First, while the pump laser is muted, a probe laser is used to scan the transmission spectrum of the Raman mode exhibiting a typical doublet, i.e., the two standing-wave supermodes arising from the intrinsic Rayleigh scattering. As the scatterer is moved closer to the cavity, the splitting of the Raman mode changes, indicating an increased or decreased intrinsic coupling strength (Fig. 4a). For each of these configurations, we excite the pump mode to study Raman lasing and the chirality of the laser associated with it (Fig. 4b). It is found that the symmetry-breaking threshold experiences an evident increase with larger intrinsic coupling strength. Moreover, the dependence of the threshold power on the mode splitting exhibits a positive linear correlation (Fig. 4c) consistent with the theoretical prediction. In this way we can thus dynamically switch between bi-directional and chiral emission of the laser by adjusting the intrinsic linear coupling that controls the threshold of breaking symmetry.

**Fig. 5:**
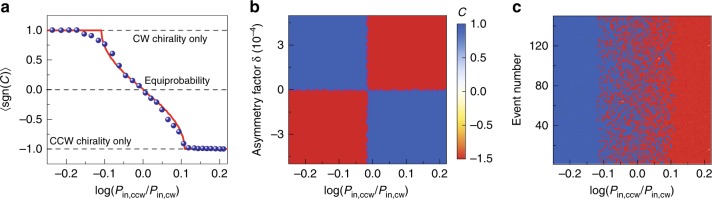
Switch of the chiral microlaser. **a** Sign of chirality averaged over different scans ($\langle {\rm{sgn}}({\mathcal{C}})\rangle$) is tuned by the bias in the pump intensities, as quantified here by $\mathrm{log}\,({P}_{{\rm{in}},{\rm{cw}}}/{P}_{{\rm{in}},{\rm{ccw}}})$. The experimental (theoretical) results are shown by the blue dots (red curve). **b** Dependence of the Raman laser chirality on the bi-directional pump ratios and the asymmetry gain factor *δ*. Here the phase difference between the two-sided inputs is set to be π. **c** Theoretical prediction for the emergence of laser chirality with the same total pump power and different bi-directional pump ratios. For every pump ratio, 150 chiral lasing events are calculated with random phase differences between the bi-directional inputs. Averaging over these random phases results in the red curve in **a**.

Furthermore, we show that a deterministic and reconfigurable direction of the chirality ${\mathcal{C}}$ can be obtained by adjusting the ratio of the CW to CCW pump intensity *P*_in,cw_*/P*_in,ccw_. In the experiment, the pump powers from two sides are independently controlled by variable optical attenuators, and each experimental event of a realized laser chirality is analyzed when the power is above the symmetry-breaking threshold. For each pump intensity ratio, more than 20 symmetry-broken-laser events are recorded during a period of time, which are then averaged to showcase the preference in the direction of laser chirality. Figure 5a shows the dependence of the chirality direction on the imbalance in the two-sided pumping, averaged over all the chiral laser events. Here the sign of ${\mathcal{C}}\in [-1,1]$ describes the direction of chirality in a specific Raman laser. When the pump intensities are balanced, the random emergence of the CW and CCW chiral laser is equiprobable with an averaged sign of chirality being close to zero. Once the ratio in pump intensities becomes slightly unbalanced, the directions of the chiral emissions exhibit a preference but are still not deterministic. When the CW-CCW (CCW-CW) ratio of pump intensities further increases to be over 1.4:1, the unidirectional Raman emission becomes deterministic in CW- (CCW-) direction, whereby we achieve the desired and full control over the direction of the chiral laser emission. In the deterministic chirality regime, the laser preferentially emits into the direction, along which the dominant pump light propagates.

This phenomenon is mainly attributed to the slightly asymmetric Raman gain *G*_R,m_ and ${G}_{{\rm{R}},{\rm{m}}^{\prime} }$ brought by the directional pump within a non-ideal medium^39^. Besides, the occurrence of the deterministic chiral direction is attributed to the pump-induced chiral performance, i.e., the intensity and phase differences between the two-sided inputs can control the emergence of chiral states as well as the randomness. The asymmetric Raman gain can be described as ${G}_{{\rm{R}},{\rm{m}}}=(1+\delta ){G}_{{\rm{R}},{\rm{m}}^{\prime} }$ with the asymmetry factor *δ*. Through the theoretical calculation (see Supplementary Note 2), it can be found that even though the Raman gain from two directions are only different slightly, the distinct chirality behavior appears, as shown in Fig. 5b. For example, as for a positive asymmetry factor *δ* of 10^−4^ (for which the forward Raman gain is slightly stronger than the backward gain), the chirality direction of the Raman laser is completely random under the identical CW and CCW pump intensity. However, once the pump intensities are not balanced any more, the lasing direction becomes dependent on the dominant direction of the pump, which is absent for the symmetric Raman gain. For a negative value of *δ*, the inverse phenomenon is obtained. To accurately model the actual situation in the experiment we take into account that the phase of the input light fluctuates due to mechanical or thermal perturbations. Correspondingly, for every different pump ratio, we evaluate the emergence of the laser chirality using equation (1) with 150 random phase differences between the two-sided inputs and plot the statistical diagram of the chiral events in Fig. 5c. The dependence of the chirality sign on the input ratio is then averaged over these realizations, $\langle {\rm{sgn}}({\mathcal{C}})\rangle$, resulting in the red curve of Fig. 5a that shows good agreement with the experimental data.

## Discussion

In summary, we have demonstrated a reconfigurable symmetry-broken microlaser in a symmetric ultrahigh-Q WGM microcavity, which is free from any delicate structure design. Such a chiral emitting Raman laser benefits from the Kerr nonlinearity and proves the long-standing prediction of SSB in an active microcavity induced by third-order nonlinearity. Note that the directional laser can also be obtained by other mechanisms in semiconductors, depending on special properties of particular materials. For example, a unidirectional Raman laser can emerge in a silicon ring resonator through the asymmetric nonlinear loss^40^ or the on-negligible longitudinal components of the optical field^41^. Besides, the unidirectional lasing was also reported in III-V compound semiconductor ring lasers using self and cross-gain saturation effects from the carriers^42,43^. However, the limited Q factors of these systems would strongly restrict further development and applications with ultralow power consumption. Our results extend nonlinear symmetric optics from the centimeter-scale to on-chip micro-structures and, more importantly, from waveguides to microresonators. The mechanism behind our observations can be directly expanded to other optical microstructures and materials, where the Raman gain could also be replaced by other nonlinear processes including those based on parametric amplification, quantum dots and rare-earth ions.

## Methods

### Lasing condition and pump clamping effect

When a cavity mode is lasing, the dissipation of the mode is completely compensated by the gain. Considering that the actual Raman mode is a standing-wave supermode rather than an uncoupled propagating wave mode, we employ the coupled-mode equation with the supermode basis *a*_1_–*a*_2_ to investigate the lasing condition, and Eq. (1) is rewritten as,

2 $$\frac{{\rm{d}}{a}_{\mu }}{{\rm{d}}t}=-\frac{{\kappa }_{\mu }}{2}{a}_{\mu }+{G}_{{\rm{R}}}| {a}_{{\rm{P}}}{| }^{2}{a}_{\mu },$$

where *κ*_*μ*_ is the decay rate of the corresponding standing-wave Raman mode. Note that here the Kerr nonlinearity term of the Raman mode in Eq. (1) is neglected, because the Raman light is extremely weak below the lasing threshold. As the pump intensity ∣*a*_P_∣^2^ increases from zero, the total decay rates of both the low- and high-frequency standing-wave mode keep decreasing. When the increasing gain is strong enough to compensate for the loss of one standing-wave mode with the lower decay rate ${\kappa }_{\min }$, the cavity mode reaches the lasing threshold. On the other hand, a stable lasing process requires that the net gain (or loss) should remain zero, resulting in the pump intracavity power being clamped at the threshold $| {a}_{{\rm{P}}}{| }^{2}={\kappa }_{\min }/(2{G}_{{\rm{R}}})$. Therefore, only one mode can survive and lase due to the mode competition and pump clamping^33^.

### Potential well of the system and the symmetry-breaking threshold

For the stable lasing with balanced gain and loss, we can promote the amplitudes *a*_*μ*_ to bosonic operators, e.g., ${a}^{* }\to \sqrt{\hslash {\omega }_{{\rm{R}}}}{a}^{\dagger }$, and construct a Hamiltonian for the system,

3 $$H	= -\sum _{m={\rm{cw}},{\rm{ccw}}} [\hslash {g}_{0}{a}_{m^{\prime} }^{\dagger }{a}_{m}+\frac{1}{2}{\hslash }^{2}{K}_{{\rm{R}}}{\omega }_{{\rm{R}}}({a}_{m}^{\dagger }{a}_{m}^{\dagger }{a}_{m}{a}_{m}+{a}_{m}^{\dagger }{a}_{m^{\prime} }^{\dagger }{a}_{m}{a}_{m^{\prime} }\\ 	\ \;\,+\,{a}_{m}^{\dagger }{a}_{m^{\prime} }^{\dagger }{a}_{m^{\prime} }{a}_{m})].$$

By employing the Bloch sphere parameters, ${a}_{{\rm{cw}}}=A{{\rm{e}}}^{{\rm{i}}\alpha }\cos (\theta /2)$ and ${a}_{{\rm{ccw}}}=A{{\rm{e}}}^{{\rm{i}}\alpha }\sin (\theta /2){{\rm{e}}}^{{\rm{i}}\varphi }$ will be used from here on^29^, where *A*e^*iα*^ is the total complex amplitude for the Raman field, 0 ≤ *θ* ≤ *π* represents the composition of the propagating waves, and 0 ≤ *φ* < 2*π* is the relative phase. The chirality can be characterized by ${\mathcal{C}}=(| {a}_{{\rm{cw}}}{| }^{2}-| {a}_{{\rm{ccw}}}{| }^{2})/(| {a}_{{\rm{cw}}}{| }^{2}+| {a}_{{\rm{ccw}}}{| }^{2})\equiv \cos \theta$. In this case, the behavior of symmetry breaking can also be made clear via the classical Hamiltonian,

4 $$H=-\frac{{g}_{0}}{{\omega }_{{\rm{R}}}}{A}^{2}\sin \theta \cos \varphi -\frac{{K}_{{\rm{R}}}}{4{\omega }_{{\rm{R}}}}{A}^{4}(2+{\sin }^{2}\theta ),$$

from which the equation of motion of the chirality is derived,

5 $$\ddot{{\mathcal{C}}}=-{g}_{0}^{2}{\mathcal{C}}+\frac{{K}_{R}{A}^{2}}{2}{\mathfrak{C}}\sqrt{{g}_{0}^{2}(1-{{\mathcal{C}}}^{2})-{\dot{{\mathcal{C}}}}^{2}},$$

where dots above variables represent derivatives with respect to time. For steady states requiring $\dot{{\mathcal{C}}}=0$, and we can rewrite the equation as

6 $$\frac{1}{{g}_{0}^{2}\sqrt{1-{{\mathcal{C}}}^{2}}}\ddot{{\mathcal{C}}}=-\frac{\partial V}{\partial {\mathcal{C}}},\ \ V=1-\sqrt{1-{{\mathcal{C}}}^{2}}-\frac{{K}_{{\rm{R}}}{A}^{2}}{4{g}_{0}}{{\mathcal{C}}}^{2}$$

By analogy of classical mechanics $m\ddot{x}=-\partial V/\partial x$, the steady state can only be found at the minimum of this potential of chirality *V*. Particularly, the threshold point *A*^2^ = 2*g*_0_*/K*_R_ is indicated by a zero second-order derivative, resulting the symmetry breaking threshold of output Raman power being ${P}_{{\rm{R}},{\rm{th}}}=| \sqrt{{\kappa }_{{\rm{in}},{\rm{R}}}}A{| }^{2}=(2{\kappa }_{{\rm{in}},{\rm{R}}}{g}_{0})/{K}_{{\rm{R}}}$, where *κ*_in,R_ is the fiber-cavity coupling rate for the Raman mode.

## Supplementary information

Supplementary Information

## Data Availability

The data that support the plots within this paper and other findings of this study are available from the corresponding author upon reasonable request. Source data for Figs. 2–5 are available at 10.6084/m9.figshare.11439357.

## Code availability

The codes that support the plots within this paper and other findings of this study are available from the corresponding author upon reasonable request.
