# Supplementary material for: Reconfigurable symmetry-broken laser in a symmetric microcavity
Source: Nat Commun. 2020 Feb 28;11:1136. doi: 10.1038/s41467-020-14861-5 (PMC7048813; doi:10.1038/s41467-020-14861-5)
Supplement: Supplementary file 1 — Supplementary Information [file 41467_2020_14861_MOESM1_ESM.pdf]

Supplementary Information:

Reconfigurable symmetry-broken laser in a symmetric microcavity

Cao et al.

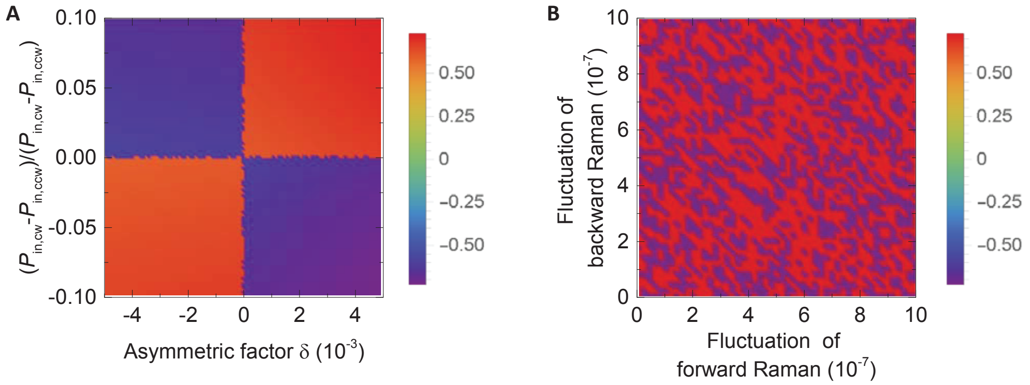

Supplementary Figure 1. Chirality of the Raman laser. **A**, Dependence of the Raman chirality on the ratio of powers in the two pump modes and the asymmetric gain factor. Parameters: $g_0/2\pi = 7$ MHz, $\kappa/2\pi = 2.6$ MHz, $K_\mathrm{R}/2\pi = 1.5$ MHz, $G_\mathrm{R}/2\pi = 0.02$ MHz, $\omega_\mathrm{P}/2\pi = 1550$ nm, $\omega_\mathrm{R}/2\pi = 1650$ nm, $P_\mathrm{in,total} = 2.5$ mW. **B**, Dependence of the Raman chirality on the initial condition of the two Raman modes. The parameters are the same as that in **A** but with the identical input power from the two directions.

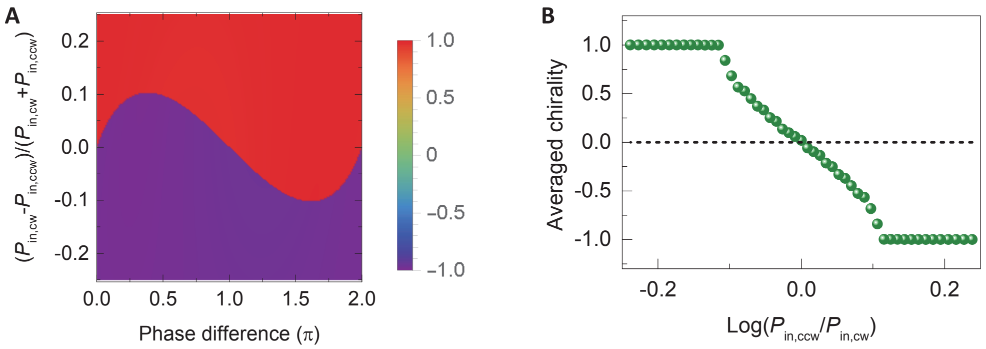

Supplementary Figure 2. **A**, Dependence of the pump chirality on the ratio of the two pump powers and their phase difference. Parameters: $g_\mathrm{P}/2\pi = 5$ MHz, $K_\mathrm{P}/2\pi = 1.5$ MHz, $\delta = 0.002$, $P_\mathrm{in,total} = 2$ mW. **B**, Dependence of the Raman chirality on the ratio of pump powers with phase difference averaged.

Supplementary Note 1: Nonlinear coupled-mode equation

We start from the Maxwell equation for the pump (Raman) electric field $\mathbf{E}_\mathrm{P}$ ($\mathbf{E}_\mathrm{R}$) in a nonlinear resonator,

$$\nabla \times (\nabla \times \mathbf{E}_\mathrm{F}) - \frac{n_\mathrm{F}^2}{c^2}\frac{\partial^2 \mathbf{E}_\mathrm{F}}{\partial t^2} = -\frac{1}{\varepsilon_0 c^2}\frac{\partial^2 \mathbf{P}_\mathrm{NL,F}}{\partial t^2} \tag{1}$$

where $\mathrm{F} = \mathrm{P}, \mathrm{R}$ corresponds to pump and Raman fields (similarly hereinafter), n_F is the refractive index of the material, ε_0 denotes the vacuum dielectric constant, and c stands for the speed of light in vacuum. Now we focus on the Raman-related third-order nonlinear polarization $\mathbf{P}_\mathrm{NL,R}$, which consists of both pump and Raman contributions [1],

$$\mathbf{P}_\mathrm{NL,R} = 6\varepsilon_0\chi^{(3)}_\mathrm{PR}(\mathbf{E}_\mathrm{P}\cdot\mathbf{E}^*_\mathrm{P})\mathbf{E}_\mathrm{R} + 3\varepsilon_0\chi^{(3)}_\mathrm{RR}(\mathbf{E}_\mathrm{R}\cdot\mathbf{E}^*_\mathrm{R})\mathbf{E}_\mathrm{R}, \tag{2}$$

where $\chi^{(3)}_\mathrm{PR}$ ($\chi^{(3)}_\mathrm{RR}$) is the third-order nonlinear susceptibility for the process $\omega_\mathrm{R} = \omega_\mathrm{R} + \omega_\mathrm{P} - \omega_\mathrm{P}$ ($\omega_\mathrm{R} = \omega_\mathrm{R} + \omega_\mathrm{R} - \omega_\mathrm{R}$), and ω_P (ω_R) is the angular frequency of the pump (Raman) field. Both $\chi^{(3)}$ terms contain Kerr effects, and the $\chi^{(3)}_\mathrm{PR}$ term also contains Raman gain for the Raman field, which can be expressed using the Kerr nonlinear index $n^{(2)}$ and the bulk Raman gain coefficient g_R:

$$\mathbf{P}_\mathrm{NL,R} = n_\mathrm{P} n_\mathrm{R}\varepsilon_0^2 c(4n^{(2)}_\mathrm{PR} - i\frac{c}{2\omega_\mathrm{R}}g_\mathrm{R})(\mathbf{E}_\mathrm{P}\cdot\mathbf{E}^*_\mathrm{P})\mathbf{E}_\mathrm{R} + 4n_\mathrm{R}^2\varepsilon_0^2 c n^{(2)}_\mathrm{RR}(\mathbf{E}_\mathrm{R}\cdot\mathbf{E}^*_\mathrm{R})\mathbf{E}_\mathrm{R}, \tag{3a}$$

$$n^{(2)}_\mathrm{PR} = \frac{3}{2n_\mathrm{P} n_\mathrm{R}\varepsilon_0 c}\mathrm{Re}\chi^{(3)}_\mathrm{PR}, \tag{3b}$$

$$n^{(2)}_\mathrm{RR} = \frac{3}{4n_\mathrm{R}^2\varepsilon_0 c}\mathrm{Re}\chi^{(3)}_\mathrm{RR}, \tag{3c}$$

$$g_\mathrm{R} = -\frac{12\omega_\mathrm{R}}{\varepsilon_0 n_\mathrm{P} n_\mathrm{R} c^2}\mathrm{Im}\chi^{(3)}_\mathrm{PR}. \tag{3d}$$

Coupling between the counter-propagating waves will lift the degeneracy and results in two standing-wave modes for both pump and Raman fields. Now we assume the electric fields of these modes are

$$\mathbf{E}_{\mathrm{F},\mu}(\mathbf{r},t) = a_{\mathrm{F},\mu}\mathbf{A}_{\mathrm{F},\mu}(\mathbf{r})e^{-i\omega_\mathrm{F} t}, \tag{4}$$

where $\mu = 1, 2$ stands for the low- and high-frequency standing-wave modes. The $\mathbf{A}_{\mathrm{F},\mu}(\mathbf{r})$ is the spatial part of the modes and $a_{\mathrm{F},\mu}(t)$ is the field amplitude, both properly normalized separately for pump and Raman fields, with

$$\delta_{\mu\nu} = \frac{1}{2}\int \varepsilon_\mathrm{F}(\mathbf{r})(\mathbf{A}_{\mathrm{F},\mu}(\mathbf{r})\cdot\mathbf{A}^*_{\mathrm{F},\nu}(\mathbf{r}))d^3\mathbf{r}, \tag{5a}$$

$$|a_{\mathrm{F},\mu}|^2 = \frac{1}{2}\int \varepsilon_\mathrm{F}(\mathbf{r})(\mathbf{E}_{\mathrm{F},\mu}(\mathbf{r},t)\cdot\mathbf{E}^*_{\mathrm{F},\mu}(\mathbf{r},t))d^3\mathbf{r}, \tag{5b}$$

where $|a_{\mathrm{F},\mu}|^2$ corresponds to the total energy of the mode and $\varepsilon_{\mathrm{F}}(\mathbf{r})$ the distribution of the dielectric constant.

Using the slowly-varying envelope approximation $|\partial^2 a/\partial t^2| \ll \omega|\partial a/\partial t|$, we expand the Raman part of Supplementary Equation 1 with these two Raman modes and two pump modes to get

$$\begin{aligned}\frac{da_\mu}{dt}\mathbf{A}_{\mathrm{R},\mu} &= (-1)^{\mu+1} i g_0 a_\mu \mathbf{A}_{\mathrm{R},\mu} \\ &+ \frac{n_{\mathrm{P}}}{n_{\mathrm{R}}}\varepsilon_0 c(2i\omega_{\mathrm{R}} n^{(2)}_{\mathrm{PR}} + \frac{c}{4} g_{\mathrm{R}})[(a_{\mathrm{P},\sigma}\mathbf{A}_{\mathrm{P},\sigma})\cdot(a_{\mathrm{P},\rho}\mathbf{A}_{\mathrm{P},\rho})^*](a_\nu \mathbf{A}_{\mathrm{R},\nu}) \\ &+ 2i\omega_{\mathrm{R}}\varepsilon_0 c n^{(2)}_{\mathrm{RR}}[(a_\sigma \mathbf{A}_{\mathrm{R},\sigma})\cdot(a_\rho \mathbf{A}_{\mathrm{R},\rho})^*](a_\nu \mathbf{A}_{\mathrm{R},\nu}),\end{aligned} \tag{6}$$

where repeated Latin indices are summed over, and all subscripts "R" in the field amplitudes are omitted from here on. The coupling strength g_0 between the Raman propagating waves by the backscattering leads to a splitting of $2g_0$ and manifests itself as the detuning term. Now we take advantage of the orthogonality of modes and left-multiply with $\frac{1}{2}\int \varepsilon_{\mathrm{R}}(\mathbf{r})\mathbf{A}^*_{\mathrm{R},\mu} d^3\mathbf{r}$ to find the equation of motion,

$$\begin{aligned}\frac{da_\mu}{dt} &= (-1)^{\mu+1} i g_0 a_\mu \\ &+ \frac{1}{n_{\mathrm{P}} n_{\mathrm{R}}}(4i\omega_{\mathrm{R}} c n^{(2)}_{\mathrm{PR}} + \frac{c^2}{2} g_{\mathrm{R}})\frac{f_{\mathrm{PR},\mu\nu\rho\sigma}}{V_{\mathrm{PR}}} a_\nu a^*_{\mathrm{P},\rho} a_{\mathrm{P},\sigma} \\ &+ \frac{1}{n^2_{\mathrm{R}}} 4i\omega c n^{(2)}_{\mathrm{RR}} \frac{f_{\mathrm{RR},\mu\nu\rho\sigma}}{V_{\mathrm{RR}}} a_\nu a^*_\rho a_\sigma,\end{aligned} \tag{7}$$

where the second-order mode volume and the intermodal coupling factor are given as

$$V_{\mathrm{FR}} = \left[\int (\frac{\varepsilon_0 n^2_{\mathrm{F}}}{2}\mathbf{A}^*_{\mathrm{F,cw}}\cdot\mathbf{A}_{\mathrm{F,cw}})(\frac{\varepsilon_0 n^2_{\mathrm{R}}}{2}\mathbf{A}^*_{\mathrm{R,cw}}\cdot\mathbf{A}_{\mathrm{R,cw}}) d^3\mathbf{r}\right]^{-1}, \tag{8a}$$

$$f_{\mathrm{FR},\mu\nu\rho\sigma} = V_{\mathrm{FR}} \times \int (\frac{\varepsilon_0 n^2_{\mathrm{F}}}{2}\mathbf{A}^*_{\mathrm{F},\rho}\cdot\mathbf{A}_{\mathrm{F},\sigma})(\frac{\varepsilon_0 n^2_{\mathrm{R}}}{2}\mathbf{A}^*_{\mathrm{R},\mu}\cdot\mathbf{A}_{\mathrm{R},\nu}) d^3\mathbf{r}. \tag{8b}$$

Here we have used the spatial parts of a propagating wave $\mathbf{A}_{\mathrm{F,cw}} \equiv (\mathbf{A}_{\mathrm{F},1} - i\mathbf{A}_{\mathrm{F},2})/\sqrt{2}$ in the mode volumes for later convenience. Similar derivations of the nonlinear coupled-mode equation can also be found in Refs. [2–4].

For a circular cavity, we have $\mathbf{A}_{\mathrm{F},1} \propto \cos m_{\mathrm{F}}\phi$ and $\mathbf{A}_{\mathrm{F},2} \propto \sin m_{\mathrm{F}}\phi$, where m_{F} is the angular momentum number, ϕ is the azimuthal coordinate of the cavity, and the same polarization is assumed for clockwise (CW) and counter-clockwise (CCW) waves. Therefore, by integrating in the ϕ direction, the intermodal coupling factor can be calculated explicitly

as

$$f_{\mathrm{PR},\mu\nu\rho\sigma} = \delta_{\mu\nu}\delta_{\rho\sigma}, \tag{9a}$$

$$f_{\mathrm{RR},\mu\nu\rho\sigma} = \frac{1}{2}(\delta_{\mu\nu}\delta_{\rho\sigma} + \delta_{\mu\sigma}\delta_{\nu\rho} + \delta_{\mu\rho}\delta_{\nu\sigma}). \tag{9b}$$

Changing into the CW-CCW basis with

$$a_{\mathrm{F,cw}} = \frac{1}{\sqrt{2}}(a_{\mathrm{F},1} + ia_{\mathrm{F},2}), \tag{10a}$$

$$a_{\mathrm{F,ccw}} = \frac{1}{\sqrt{2}}(a_{\mathrm{F},1} - ia_{\mathrm{F},2}), \tag{10b}$$

the coupled-mode equations now read

$$\frac{da_m}{dt} = ig_0 a_{m'} + (G_\mathrm{R} + iK_\mathrm{P})|a_\mathrm{P}|^2 a_m + iK_\mathrm{R}(|a_m|^2 + 2|a_{m'}|^2)a_m, \tag{11}$$

where $|a_\mathrm{P}|^2 = |a_{\mathrm{P},1}|^2 + |a_{\mathrm{P},2}|^2$ is the total pump intensity, m and m' ($m \neq m'$) stands for CW and CCW, and we have defined the coefficients as

$$G_\mathrm{R} = \frac{c^2}{2n_\mathrm{P} n_\mathrm{R} V_\mathrm{PR}} g_\mathrm{R}, \tag{12a}$$

$$K_\mathrm{P} = \frac{4\omega_\mathrm{R} c}{n_\mathrm{P} n_\mathrm{R} V_\mathrm{PR}} n^{(2)}_\mathrm{PR}, \tag{12b}$$

$$K_\mathrm{R} = \frac{4\omega_\mathrm{R} c}{n_\mathrm{R}^2 V_\mathrm{RR}} n^{(2)}_\mathrm{RR}. \tag{12c}$$

For an actual system where dissipation is considered and a waveguide is used to couple out the Raman modes, the coupled-mode equations acquire additional loss and detuning terms,

$$\frac{da_m}{dt} = (i\Delta + iK_\mathrm{P}|a_\mathrm{P}|^2 - \frac{\kappa}{2})a_m + ig_0 a_{m'} + G_\mathrm{R}|a_\mathrm{P}|^2 a_m + iK_\mathrm{R}(|a_m|^2 + 2|a_{m'}|^2)a_m, \tag{13}$$

where Δ is the detuning with respect to the reference frequency of original Raman mode, and κ_m is the decay rate. Switching to the resonance rotating frame in which the Raman field has zero total detuning, the coupled-mode equations read

$$\frac{da_m}{dt} = ig_0 a_{m'} - \frac{\kappa}{2} a_m + G_\mathrm{R}|a_\mathrm{P}|^2 a_m + iK_\mathrm{R}(|a_m|^2 + 2|a_{m'}|^2)a_m. \tag{14}$$

Supplementary Note 2: Asymmetric gain effect

In this section, we provide an explanation for the pump direction-dependent Raman chirality. As shown in Supplementary Equation 14, the pump state (i.e. CW and CCW components

of pump) does not appear in the equation because the Raman process is incoherent and does not distinguish between different momenta of pump photons. This property has been well recognized in nonlinear optics as the strengths of the forward and backward stimulated Raman scattering are equal in an ideal medium [1]. Nevertheless, previous studies also reported on forward-dominating stimulated Raman scattering, which is attributed to effects, like a difference in the cross section, self-focusing effect, etc. [5]. Taking such an asymmetric scattering strength into account, we rewrite Supplementary Equation 14 in the general form,

$$\frac{\mathrm{d}a_{\mathrm{R,m}}}{\mathrm{d}t} = -\frac{\kappa}{2}a_{\mathrm{R,m}} + (G_{\mathrm{R,m}}|a_{\mathrm{P,m}}|^2 + G_{\mathrm{R,m'}}|a_{\mathrm{P,m'}}|^2)a_{\mathrm{R,m}} + ig_0 a_{\mathrm{R,m'}} + iK_{\mathrm{R}}(|a_{\mathrm{R,m}}|^2 + 2|a_{\mathrm{R,m'}}|^2)a_{\mathrm{R,m}}, \tag{15}$$

incorporating the evolution of pump modes,

$$\frac{\mathrm{d}a_{\mathrm{P,m}}}{\mathrm{d}t} = -\frac{\kappa_0 + \kappa_{\mathrm{in}}}{2}a_{\mathrm{P,m}} - \frac{\omega_P}{\omega_R}(G_{\mathrm{R,m}}|a_{\mathrm{R,m}}|^2 + G_{\mathrm{R,m'}}|a_{\mathrm{R,m'}}|^2)a_{\mathrm{P,m}} + i\Delta a_{\mathrm{P,m}} + ig_{\mathrm{P}}a_{\mathrm{P,m'}} + iK_{\mathrm{P}}(|a_{\mathrm{P,m}}|^2 + 2|a_{\mathrm{P,m'}}|^2)a_{\mathrm{P,m}} + \sqrt{\kappa_{\mathrm{in}}}a_{\mathrm{in}}, \tag{16}$$

where $G_{\mathrm{R,m(m')}}$ denotes the Raman gain coefficient from the m (m')-direction pump, and $G_{\mathrm{R,m}} = (1+\delta)G_{\mathrm{R,m'}}$ with the asymmetric factor δ; κ_0 (κ_{in}) is the intrinsic (coupling) loss of the pump mode, and Δ is the detuning between the input and the cavity resonant frequency; a_{in} is the input intensity with the power of $|a_{\mathrm{in}}|^2$. Note that here the linear coupling (with the strength g_{P}) and the optical Kerr effect (with the coefficient K_{P}) of the pump counter-propagating modes are also involved as a general model of the interaction.

When the high-frequency pump mode (out of phase) is excited and the input is beyond the chirality threshold, the pump intracavity field exhibits a chiral propagation with unbalanced CW and CCW intensities of the pump mode, as revealed in Ref. [4]. As the input power keeps increasing and reaches the laser threshold, a Raman laser is excited with balanced bi-directional emission initially. When the laser intensity exceeds the chirality threshold, the unidirectionality of the Raman laser emerges. Even though the Raman gain coefficients from two directions are only slightly different, distinct chirality behavior appears, as shown by the theoretical results in Supplementary Figure 1. For example, for a positive asymmetric factor δ of 0.001 (for which the forward SRS is slightly stronger than the backward SRS), when the CW and CCW pump intensities are strictly identical, the chirality direction of the Raman laser is completely random without any preferred direction, as shown in Supplementary Figure 1B. However, once the pump intensities are not equal, the chirality direction becomes

dependent on the dominant direction of the pump. For a negative value of δ, the inverse phenomenon is obtained. In short, the nature of the pump direction-dependent Raman chirality is that the linear coupling is canceled out but the Kerr effect of the Raman laser, and the asymmetric gain is utilized by the unbalanced pump field. Here the chirality of the pump field could also be obtained directly by the unbalanced input in absence of the linear coupling of the pump mode.

Furthermore, we now consider the phase fluctuation of the input light as present in our experiment. Under bi-directional input, the laser is divided into two paths, i.e., $a_{\mathrm{in},1} = A_1 e^{i\omega_{\mathrm{in}}t+\phi_1(t)}$ and $a_{\mathrm{in},2} = A_2 e^{i\omega_{\mathrm{in}}t+\phi_2(t)}$, where A_i and ω_{in} are the amplitude and frequency of the input light, respectively. $\phi_i(t)$ is the additional phase induced by mechanical perturbations or thermal fluctuations in each path from the tunable diode laser to the cavity. Therefore, the bidirectional inputs are subject to a drift of the phase difference extending over time from 0 to 2π. With these different phases of the input, the chirality of the pump cavity field is obtained through the theoretical calculations as shown in Supplementary Figure 2**A**. We further analyze the dependence of the averaged chirality on the input ratio, as plotted in Supplementary Figure 2**B**, which is similar to figure 4 in the main text. It is found that when the inputs are balanced, the emergence of the CW and CCW chiral pump field is equiprobable with an averaged chirality being very close to zero. Once the ratio in input intensities becomes unbalanced but their differences remain small, the directions of the chiral pump field exhibit a preference but are still indeterministic. When the CW-CCW (CCW-CW) input intensities ratio further increases, the unidirectional pump field is deterministic in CW- (CCW-) direction. Incorporating the relation between the chirality of the pump and the Raman laser as mentioned above, the behavior of Supplementary Figure 2**B** can give rise to the phenomenon shown in figure 4 of the main text. The preference in direction of the laser chirality is thus positively correlated with the dominance of the input direction.

Supplementary References

[1] Boyd, R. W. Nonlinear Optics (Academic, 2003).

[2] Yang, X. & Wong, C. W. Coupled-mode theory for stimulated Raman scattering in high-Q/V_m silicon photonic band gap defect cavity lasers. *Opt. Express* **15**, 4763-4780 (2007).

[3] Chembo, Y. K. & Yu, N. Modal expansion approach to optical-frequency-comb generation with monolithic whispering-gallery-mode resonators. *Phys. Rev. A* **82**, 033801 (2010).

[4] Cao, Q.-T. et al. Experimental demonstration of spontaneous chirality in a nonlinear microresonator. *Phys. Rev. Lett.* **118**, 033901 (2017).

[5] Maier, M., Kaiser, W. & Giordmaine, J. A. Backward stimulated Raman scattering. *Phys. Rev.* **177** 580 (1969).
